# Analysis of the optical properties of the silvery spots on the wings of the Gulf Fritillary, *Dione vanillae*

**DOI:** 10.1038/s41598-021-98237-9

**Published:** 2021-09-29

**Authors:** Andrés Dolinko, Luisa Borgmann, Christian Lutz, Ernest Ronald Curticean, Irene Wacker, María Sol Vidal, Candela Szischik, Yidenekachew Donie, Marina Inchaussandague, Diana Skigin, Hendrik Hölscher, Pablo Tubaro, Ana Barreira

**Affiliations:** 1 CONICET, Consejo Nacional de Investigaciones Científicas y Técnicas, Buenos Aires, Argentina; 2 Facultad de Ciencias Exactas y Naturales, Departamento de Biodiversidad y Biología Experimental, Universidad de Buenos Aires, Ciudad Universitaria, Pabellón 2, C1428EHA Buenos Aires, Argentina; 3 Institute for Microstructure Technology, Karlsruhe Institute of Technology (KIT), Hermann-von-Helmholtz-Platz 1, 76344 Eggenstein-Leopoldshafen, Germany; 4 CryoEM, BioQuant, University of Heidelberg, Im Neuenheimer Feld 267, 69120 Heidelberg, Germany; 5 Facultad de Ciencias Exactas y Naturales, Departamento de Física, Grupo de Electromagnetismo Aplicado, Universidad de Buenos Aires, Ciudad Universitaria, Pabellón 1, C1428EHA Buenos Aires, Argentina; 6 Light Technology Institute (LTI), Karlsruhe Institute of Technology (KIT), Engesserstrasse 13, 76131 Karlsruhe, Germany; 7 Instituto de Física de Buenos Aires (IFIBA), CONICET, Universidad de Buenos Aires, Ciudad Universitaria, Pabellón 1, C1428EHA Buenos Aires, Argentina; 8 División de Ornitología, Museo Argentino de Ciencias, Naturales “Bernardino Rivadavia” MACN-CONICET, Av. Angel Gallardo 470, C1405DJR Buenos Aires, Argentina

**Keywords:** Nanoscale biophysics, Other photonics, Nanophotonics and plasmonics

## Abstract

The ventral face of the wings of the butterfly *Dione vanillae* is covered with bright and shiny silvery spots. These areas contain densely packed ground- and coverscales with a bright metallic appearance reflecting more than 50% of light uniformly over the visible range. Our analysis shows that this optically attractive feature is caused by the inner microstructure of the scales located in these areas. Electron microscopy of cross sections through the scales shows that upper and lower lamina, supporting trabeculae, and topping ridges can be approximated by a ‘circus tent’-like geometry. By simulating its optical properties, we show that a moderate disorder of this geometry is important for the uniform reflection of light resulting in the silvery appearance.

## Introduction

The colourfulness of nature has fascinated mankind since ancient times^1^. Colours in nature are so numerous because they serve many purposes ranging from camouflage and courtship to thermoregulation and protection against ultraviolet (UV) radiation. In many cases these colours are caused by pigments and dyes. Very bright colours in nature, however, are frequently produced by nanoscale structures alone^2–4^. In recent years scientists have become interested in such structural colours because they have several advantages compared to pigments and dyes. First, they are extremely bright and attract attention even when displayed in a colourful environment. Second, they do not fade as long as their colour-producing nanostructure is intact. Prominent examples are the blue fruits of the plants *Polia condensata*^5^ and *Margaritaria nobilis*^6^. Both are very easy to spot due to their famous brilliant blue caused by interference of a helicoidal structure. Furthermore, one can observe the original blue colour for many years (see, e.g., Fig. S1 in Ref.^5^) while most pigments and dyes are known to degrade within comparable short times (especially under UV radiation).

Among all plants and animals featuring structural colours, butterflies are most likely the *taxa* with the largest variation of structural colours. The famous blue *Morpho* butterflies^2,4,7^ are just one genus of the more than 157,000 species described in the order Lepidoptera (moths and butterflies)^8^. With the advent of new high resolution microscopy techniques like electron microscopy it became possible to image the nanostructures causing structural colour of animals and plants. As a result, scientists could correlate the nanostructure and the resulting colour of insects^9,10^, spiders^11,12^, fish^13^, bird feathers^14^, and plants^5,6^. In some cases the natural archetype served as an inspiration to develop artificial structural colours for various applications^7,15–17^.

Metallic-like surfaces of some insects with a brilliant silver- or gold-like appearance are a special case of structural colouration in nature. Already in 1977 Neville discovered that several insects produce a metallic-like appearance through broadband interference reflectors^9^. A more sophisticated structure has been recently described in the Saharan silver ant (*Cataglyphis bombycina*) which uses the broadband reflection of triangular hair-like structures densely covering its body for thermoregulation^18,19^. There are also several butterflies whose wings are partially or even completely covered with silvery scales^20–25^. The wings of the butterfly *Argyrophorus argenteus* are more or less completely covered with silvery scales. As described by Vukusic et al.^22^ this interesting colour effect is achieved by broadband diffusive reflectivity caused by multi-colour addition through a sub-micron design. A similar design has been recently reported by Wilts et al.^23^ and Liu et al.^24^ for the butterfly *Curetis acuta* which is covered with silvery scales on its ventral wing side. Again colour mixing leads to broadband silver reflection, although the detailed scale structure is different to that of *Argyrophorus argenteus*.

**Figure 1:**
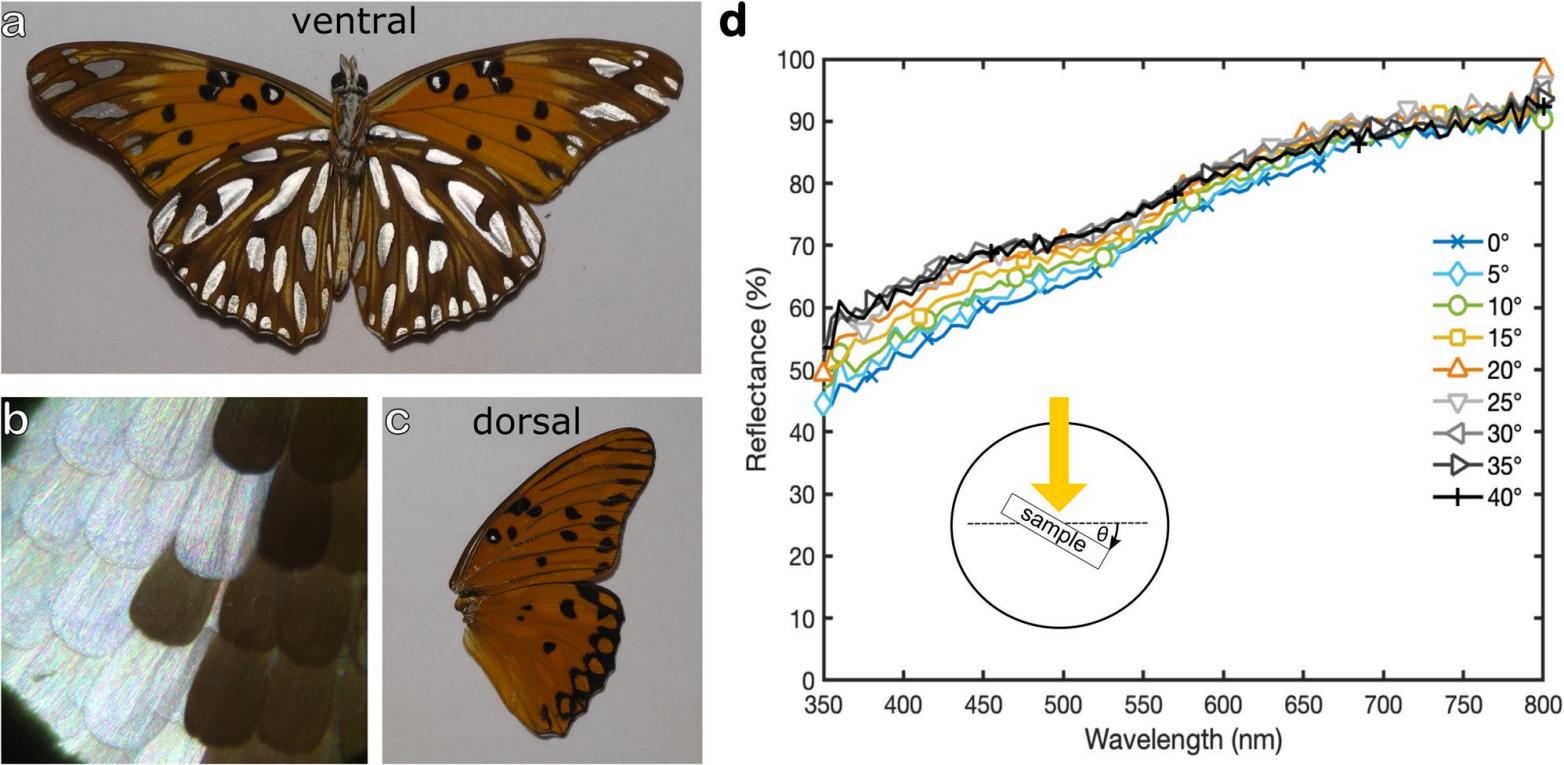
**(a)** Macroscopic colour impression of the ventral wings of the butterfly *Dione vanillae*. When viewed from the top the silvery spots shine bright and metallic. **(b)** A detail of a silvery spot consisting of silvery scales bordered by black ones. **(c)** View on the dorsal side of one wing which has only tiny silvery spots. **(d)** Reflection of a silvery spot for various tilt angles measured in an integrating sphere. The reflection is broadband and changes only slightly during the rotation of the sample. The inset describes the rotation angle.

A very widespread butterfly with silvery spots is *Dione vanillae* (Linnaeus, 1758) (previously known as *Agraulis vanillae*^26^) from the tribe *Heliconiini*. As shown in Fig. 1a, its ventral wings are covered with flashy silvery spots which explains its common Spanish name ‘espejitos’ (little mirrors). Upon changing the viewing angle, the silvery brightness of the spots changes only slightly (see Video S1). When the wings are folded only the areas with silvery spots are exposed. The distribution of *D. vanillae* spans from South to North America^27^. Interestingly, the scent scales in the wings of males—which are important for courtship^28^—were analysed already in 1877^29,30^. However, despite of its optical attractiveness, its huge dispersal area, and its early scientific description it seems that the physical origin of the metallic appearance of the silvery spots has not been examined in detail so far. Simonsen^20^ and Giraldo^21^ imaged the silver scales of *D. vanillae* by electron microscopy and compared their microstructure with that of other butterflies but no direct correlation between nanostructure and optical appearance was given.

Here, we analyse the inner structure of the silvery scales of *D. vanillae* and explore the physical origin of their metallic, silvery appearance by experiment and simulation. Utilising optical and electron microscopy we characterise the photonic structure of the scales. Optical spectroscopy is applied to measure reflection of silvery spots on the wings as well as single cover- and ground-scales. Electron microscopy is applied to characterise the inner structure of the scales. Subsequent modelling of the optical response of this inner structure demonstrates how the disordered, non-periodic arrangement of the lower and upper lamina reflects light of all visible wavelengths resulting in the silvery appearance of the scales.

## Experimental analysis of the silvery spots

The ventral wings of *D. vanillae* are densely covered with overlapping scales of different colours (mainly reddish-brown, black, and silvery). As shown in Fig. 1a,b the silvery spots shine bright while the rest of the wing appears mostly dull. No (or only a few tiny) silvery spots can be found on the dorsal forewing and the dorsal side possesses only orange and black scale patterns (Fig. 1c). Male butterflies of *D. vanillae* show a special scale formation along the veins of their forewing, bearing specialised, presumptive pheromone-disseminating scent-scales for courtship^30^. The forewing on the ventral side of *D. vanillae* is comparably colourful, it possesses black, reddish-orange, brown, and a few yellow areas as well as silvery spots at the upper forewing tip and at the leading edge. The ventral hindwing is widely covered with silvery spots, which are surrounded by black and brown areas (Fig. 1a). Additional stripe patterns of yellow scales can be found between the silvery spots. When the butterfly folds its wings only the regions with silvery spots are exposed.

The overall colour impression of the silvery spots resembles the broadband reflection of silverware or aluminium foil. The interesting colour effect is widely independent of the viewing angle. While tilting the sample the colour impression changes slightly but the silvery spots still shine bright (see Supplemental Video S1). This macroscopic observation is confirmed by optical spectroscopy recorded in an integrating sphere. As shown in Fig. 1d, the reflection spectra are quite broadband in the visible range. The reflection increases continuously from about 50% at lower wavelengths in the ultraviolet to more than 90% in the infrared. The overall spectra change only slightly during the rotation of the wing from flat (0$^\circ$) to 40$^\circ$.

Imaging the silvery spots in an optical microscope shows that they consist of silvery scales surrounded by dark ones (Fig. 1b). The photo reveals also the imbricate arrangement of scales which is typical for most butterflies. If the dark scales are overlapped by silvery ones, there is no significant colour change of the silvery scales. At a well-preserved sample position like this, only the cover scales are visible, the underlying ground scales are completely hidden. All scales are oriented in the direction of the respective wing base and the scale tips point towards the wing edges.

**Figure 2:**
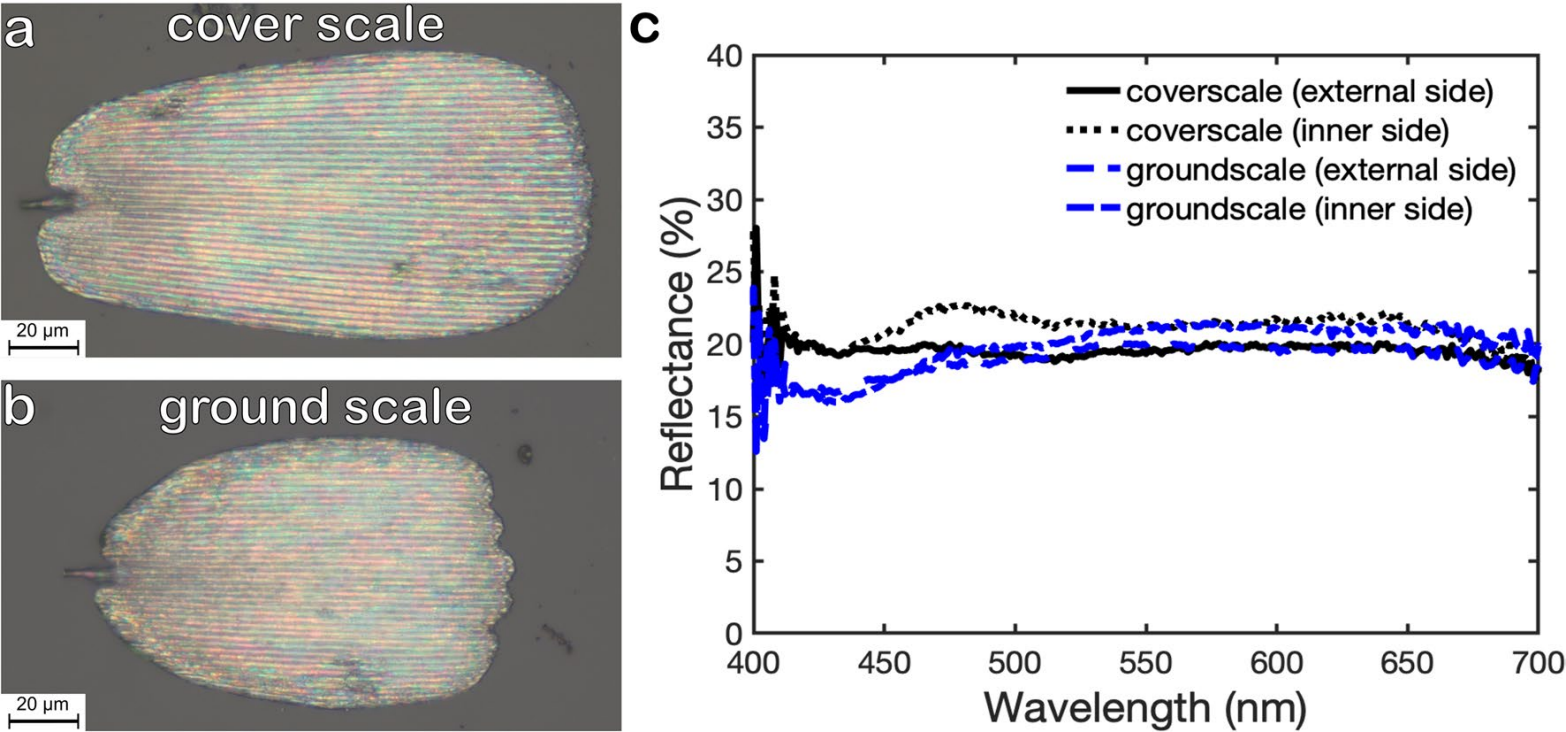
**(a)** A photo of the external side of a single silvery coverscale. **(b)** Groundscales are smaller than coverscales and have serrated tips. Both scales types, however, are covered with colourful rows shining in all colours of the visible spectrum. The ridges have very low reflection in the visible and appear dark. **(c)** Reflectance spectra of single cover and ground scales measured with a modular spectrometer in a light microscope. The data sets recorded on the external and inner side of the scales reveal a broadband reflectance of 20% in this set-up.

**Figure 3:**
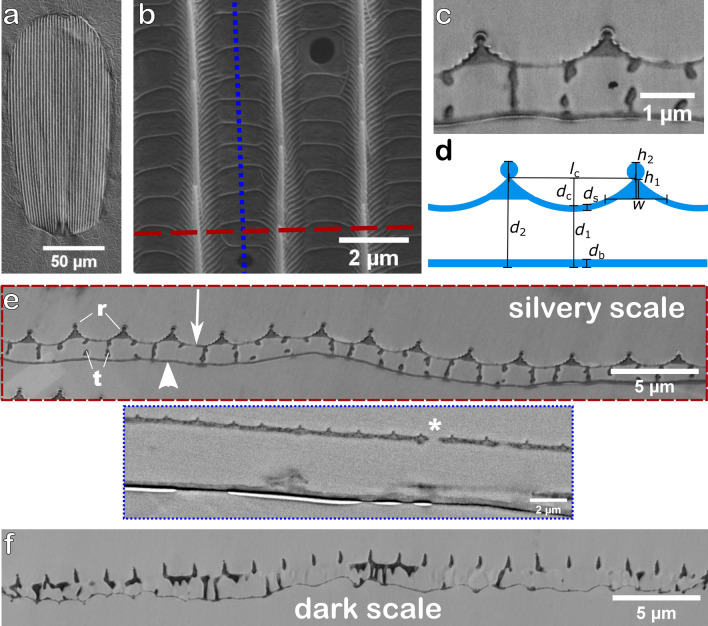
Scanning electron microscopy images of single silvery scales of *D. vanillae* and cross-sections of silvery and dark scales. **(a)** SEM image of the upper side of a silvery cover scale. **(b)** Zoom into the upper lamina of the scale shown in **(a)**. The blue dotted and red dashed line indicate the cutting directions of the sections imaged in **(c,e)**. **(c)** Electron microscopy image of a cross section along the red dashed line of a silvery scale revealing the inner structure of the scale. **(d)** ‘Circus tent’ model describing the cross-sections of the silvery scales of *D. vanillae*. Experimental values of the indicated dimensions obtained from several electron microscopy images are summarised in Tab. 1. This model is used for the simulation of a periodic structure shown in Fig. 5a. **(e)** Images of two cross-sections along the red dashed (top image—perpendicular to the ridges) and the blue dotted line (bottom image—parallel to the ridges) in **(b)**. The top image shows several repetitions of the typical assembly unit of ridges (r) on the upper lamina (arrow) which is connected by trabeculae (t) to the lower lamina (arrowhead). The bottom image demonstrates that the cavity below the membrane of the upper lamina is empty. Besides a few holes (asterisk), the upper lamina of the silvery scales is more or less completely closed. **(f)** Electron microscopy image of a dark scale. Contrary to the silvery scales, dark scales have an open upper lamina, with narrow lancet-shaped longitudinal ridges and a strongly undulated lower lamina.

Removing single scales from the wing allows to examine the different shapes of cover- and ground scales as shown in the microscopy images in Fig. 2a,b. The cover scales exhibit mostly rounded tips. We observed, however, that they become more and more serrated with increasing proximity to the edge of the wing. The cover scales are $\approx 180$ $\upmu$m in length and $\approx 80$ $\upmu$m in width. The ground scales, on the other hand, possess always serrated tips and are usually smaller. They have a typical length and width of $\approx 115$ $\upmu$m and $\approx 65$ $\upmu$m, respectively. Despite of their different size and shape, the overall colour impression of both types of scales is the same. In the microscopy image, their overall appearance is still silvery but they feature tiny colourful spots shining in all colours of the rainbow between dark parallel lines. As shown later, these dark stripes correspond to the ridges (see Fig.3e).

Broadband reflection is also observed for individual scales where no other scales influence optical features. Figure 2c shows spectra obtained on single scales. These were measured in an optical microscope with a modular spectrometer. The obtained reflection is also fairly constant in the visible range for both sides of cover- and groundscales. The reflection values, however, are lower than that for the silvery spots in Fig. 1d, only about 20% of the incoming light is reflected. This effect is partially caused by the different spectroscopy set-ups. The integrating sphere used for the measurement of the intact silvery spots collects more scattered light than that collected through a microscope objective for the single scale measurements. As demonstrated in Fig. S1, the overall reflection depends also on the actual distance between the objective lens and the sample. Furthermore, we had to use different reflectance standards for both measurements. Another possible reason for the higher reflectance on silvery spots is that the densely packed ground- and coverscales will increase the overall reflection.

Scanning electron microscopy (SEM) images of the upper silvery scale surface reveal typical, longitudinally oriented ridges (Fig. 3a). At higher magnifications in Fig. 3b it becomes apparent that the scale surface between the ridges is closed, providing a smooth layer enabling interference effects. Only few tiny circular openings towards the scale interior can be observed. This is a variation of a “generic” structure with openings between the ridges and the interconnecting cross-ribs found in many butterfly wings (see Fig. 32D in Ref.^31^). However, cross-rib structures can also be observed on the closed surface. Additionally, a multitude of micro-ribs cover the ridge side in an oblique angle down to the smooth groove surface. Viewed from above the micro-ribs form an angle of about 45$^\circ$ with respect to the ridges.

The cross-sectional view in Fig. 3c reveals the inner geometry of the scales. The longitudinal ridges exhibit a triangular shape, the lamellae between them have a circular cross section. The closed upper lamina forms a round groove and the lower lamina is very flat compared to scales of dark colour (see Fig. 3e,f for comparison). In order to quantify the structural dimensions of the silvery scales, we simplified their structure and assumed a ‘circus tent’ like model. A scheme including the relevant parameters is presented in Fig. 3d. Measurements of these structural dimensions extracted from electron microscopy images are summarised in Table 1.

**Table 1:** Averaged dimensions and their standard deviations determined from several cross-sections of silvery cover ($N = 24$) and ground scales ($N = 18$) recorded by electron microscopy. All values are in nm. All geometrical parameters are indicated in Fig. 3.

Parameter	Cover scale (nm)	Ground scale (nm)
$d_1$	987 ± 162	1135 ± 143
$d_s$	85 ± 14	94 ± 16
$d_{b}$	117 ± 13	121 ± 13
$d_2$	1655 ± 175	1783 ± 149
$h_1$	422 ± 112	388 ± 55
$h_2$	602 ± 56	560 ± 62
*w*	965 ± 97	784 ± 89
$l_c$	2033 ± 202	1775 ± 110
$d_c$	460 ± 63	477 ± 67

The distance between both laminae as well as their respective thickness is in a range where thin-film-interference effects are to be expected, explaining the microscopical multi-colour impression described above. The scale interior is only pervaded by typical pillar structures, but interestingly they are located close to the edges of the triangular ridges, leaving the space under the triangle and the groove air-filled (see Fig. 3e). Incident light can be reflected at the air-chitin interfaces and interfere. Here, it is important to note that the distances between the upper and lower lamina ($d_1$ and $d_2$) are not constant. We observed a comparable high standard deviation for the ground and cover scales, in the range of visible wavelength while the standard deviation of the thickness of the two laminae is only some tens of nanometers. As discussed below, the spatial variation of the distance between lower and upper lamina leads to a constructive interference for a range of wavelengths.

The optical impression as well as the above presented experimental analysis of the scale structure suggest that the broadband reflection of the silvery scales and spots is a structural effect caused by the microstructure of the scales. However, the classical test for structural colours is not decisive. Wetting silvery spots with a non-polar liquid like isopropanol changes the overall colour impression slightly (Fig. S2). The scales look more greenish than silvery but we did not observe a drastic change of colour as observed on *Morpho* butterflies^2,7^. We assume that the closed cavity between upper and lower lamina prevents liquids to penetrate so that possible interference effects within the scale are not altered by the liquid.

In order to learn more about the broadband reflection of the silvery scale, we analysed the reflection spectra of three different models: a simple thin-film-planar-layer-system with high variation, a periodic structure as well as the original disordered structure. The results demonstrate how the structural disorder helps to achieve broadband reflection.

## Simulation of the optical properties of silvery scales

**Figure 4:**
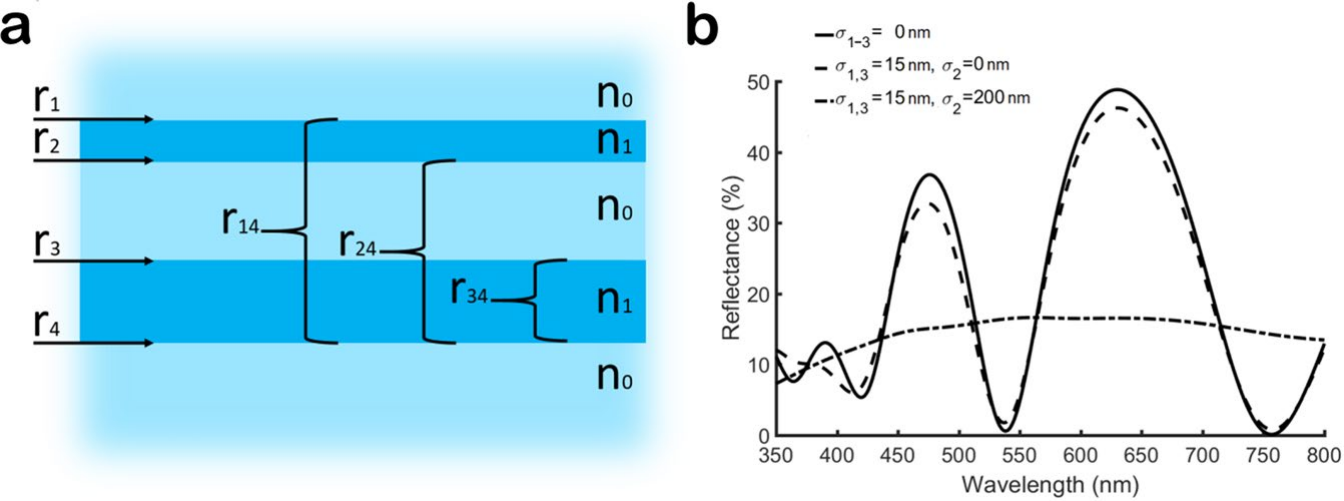
**(a)** Schematic of a simplified three-layer system representing the lower and upper lamina and the air layer in between. **(b)** Resulting reflectances for different thickness variations according to the scale dimensions revealed by electron microscopy (see Table 1). We assume an air cavity thickness of 780 nm and $\sigma _2=200$ nm. The upper and lower laminae are 90 nm and 120 nm, respectively. A standard variation of 15 nm was considered for these two chitin layers. In order to demonstrate the influence of the thickness variation we considered three cases. The solid line represents the reflectance calculated without any thickness variation while the dashed and dash-dotted lines correspond to the reflectance considering the specified standard deviations only for the two chitin or all three layers.

As pointed out already, the silvery ground and cover scales feature some structural randomness (or “disorder”) of the thickness of lower and upper laminae as well as their respective distance. A first, simple approach to investigate the influence of these variations on the reflectance is to model the upper and lower lamina in the groove region as two thin planar layers separated by an air layer as low refractive index component. Such a simplified three-layer-system is shown in Fig. 4a. A portion of the light is reflected at each interface between air and chitin, while another portion is transmitted. The overall reflectance can be calculated in the following way.

For a single thin-film, the reflectance is given by^32,33^

1 $$\begin{aligned} R(\lambda , d)=\biggl |\frac{r_1+r_2e^{-2i\delta }}{1+r_1r_2e^{-2i\delta }}\biggl |^2 \, , \end{aligned}$$

where $r_1$ is the Fresnel’s reflection coefficient at the first interface between air and chitin and correspondingly $r_2$ is the reflection coefficient at the second interface between chitin and air. The dependence of the reflectance on the wavelength $\lambda$ and the film thickness *d* is expressed by the phase delay^32^

2 $$\begin{aligned} \delta = \frac{2\pi }{\lambda } n d cos(\theta ), \end{aligned}$$

where *n* is the respective refraction index and $\theta$ is the angle of incidence.

To include a variation of the thin film thickness *d* in the reflectance calculation, a Gaussian distribution of the thickness *d* around its mean ${\overline{d}}$ with the standard deviation $\sigma$ is assumed^33^

3 $$\begin{aligned} f(d)=\frac{1}{\sqrt{2\pi \sigma ^2}} e^{-\frac{(d-{\overline{d}})^2}{2\sigma ^2}}. \end{aligned}$$

From the combination of the reflectance of a single thin film with its variation in thickness $\sigma$, the averaged reflectance can be expressed as^33^

4 $$\begin{aligned} R(\lambda )=\int _{0}^{\infty } R(\lambda ,d)f(d)dd. \end{aligned}$$

When extending this method to multi-layer systems like the one shown in Fig. 4a, further interference effects between the reflections of the various interfaces of the layers involved have to be considered. Assuming a normal angle of incidence, the reflection coefficient of the first interface can be expressed as

5 $$\begin{aligned} r_1 =\frac{n_0-n_1}{n_0+n_1}. \end{aligned}$$

Furthermore, the following relation applies for the coefficients of the remaining interfaces: $r_1$=$r_3$=$-r_2$ and $r_2$=$r_4$. According to Rouard’s method^32^ the lowest thin film layer is used as the starting point of the calculation (see Fig. 4a) and the reflected amplitude of this layer is calculated in terms of the Fresnel equations from

6 $$\begin{aligned} r_{34}=\frac{r_3+r_4e^{-2i\delta }}{1+r_3r_4e^{-2i\delta }}. \end{aligned}$$

In the following step, the next layer $r_{24}$ is considered but now $r_{34}$ is the new Fresnel reflection coefficient ((see Fig. 4a) and Eq. (7)). For the assumed three-layer-system, one more iteration is sufficient to determine $r_{14}$

7 $$\begin{aligned} r_{24}=\frac{r_2+r_{34}e^{-2i\delta }}{1+r_2r_{34}e^{-2i\delta }} r_{14}=\frac{r_1+r_{24}e^{-2i\delta }}{1+r_1r_{24}e^{-2i\delta }}. \end{aligned}$$

The resulting reflectance of the multilayer system can now be calculated by^32^

8 $$\begin{aligned} R=\bigl | r_{14} \bigl | ^2. \end{aligned}$$

We applied this method with typical values for the respective lamina thicknesses observed in the butterfly scales (Table 1). In order to consider their thickness variations, we include them in the three layer model through their respective standard deviations. Figure 4b presents the resulting reflectance spectrum for a typical thickness of 90 nm for the upper lamina and 120 nm for the lower lamina. A refractive index of 1.56 for chitin^34,35^ and a normal angle of incidence ($\theta = 0^\circ$) is assumed.

In a first step, the layer thicknesses are assumed to be constant. Therefore, their respective standard deviations are set to zero ($\sigma _1=\sigma _2=\sigma _3=0$ nm). The solid line plot in Fig. 4b represents this case. As expected, some wavelengths suffer destructive interference, while others are enhanced by constructive interference.

In a next step, we consider the relatively low variation of the chitin thin films ($\sigma _1=\sigma _3= 15$ nm, $\sigma _2=0$ nm). The dashed line in Fig. 4b corresponds to this case. The impact of this small variation on the resulting reflectance spectrum is rather limited. Distinct maxima and minima can be still observed in the reflectance spectrum.

This behaviour changes completely if the variation of the air layer is considered. Assuming a standard deviation of 200 nm for the air cavity and still including the variation of upper and lower lamina ($\sigma _1=\sigma _3=15$ nm, $\sigma _2 = 200$ nm), results in the reflectance spectrum represented by the dash-dotted line in Fig. 4b. Now, the spectrum is almost flat due to the high variation of the air cavity thickness. So, this spectrum already demonstrates that a significant disorder of the air layer thickness helps to achieve broadband reflection even for a simple three layer system.

After this demonstration of the strong influence of structural randomness on reflectance, we now analyse the optical response of the silvery scale structure in more detail. For that, we applied a photonic simulation method previously employed to compute the optical response of diverse natural structures, such as funghi^36^ and euglenoids^37^. One of its advantages is the option to define the simulation domain directly from electron microscopy images. The simulation technique is inspired by the propagation of mechanical waves along a two-dimensional array of elastically coupled particles in a *x–y* plane^38,39^. This approach can be applied to investigate the optical response of two-dimensional configurations involving dielectric materials, illuminated by transverse electric (TE) polarized light.

Again, we consider the influence of structural randomness on the reflectance spectra. Therefore, we compare a periodic with a disordered structure. For the periodic case, we simplified the cross-sectional structure to the ‘circus tent’ model shown in Fig. 3d: the cross- as well as the micro-ribs, which both have a height of approximately 50 nm and are therefore in the sub-wavelength range, are not taken into account. Concerning the internal structure the supporting pillars are omitted as there are only a few continuous pillar-structures observed in the 2D cross-sectional view and due to their arrangement away from the areas suspected to be optically important. Furthermore, the thickness of the upper and lower lamina is assumed to be constant. Typical values for the parameters indicated in the schematic in Fig. 3d and derived from the measurements presented in Table 1 are: $l_c = 2040$ nm, $h_1 = 420$ nm, $h_2 = 585$ nm, $d_s = 90$ nm, $d_{b} = 120$ nm, $d_1 = 975$ nm, $d_2 = 1650$ nm. We employed the above described photonic simulation method to compute the reflectance, transmittance and near field of this structure, for an incident Gaussian beam with an approximate width of 13 $\upmu$m considering a typical refractive index of chitin of 1.56^34,35^.

**Figure 5:**
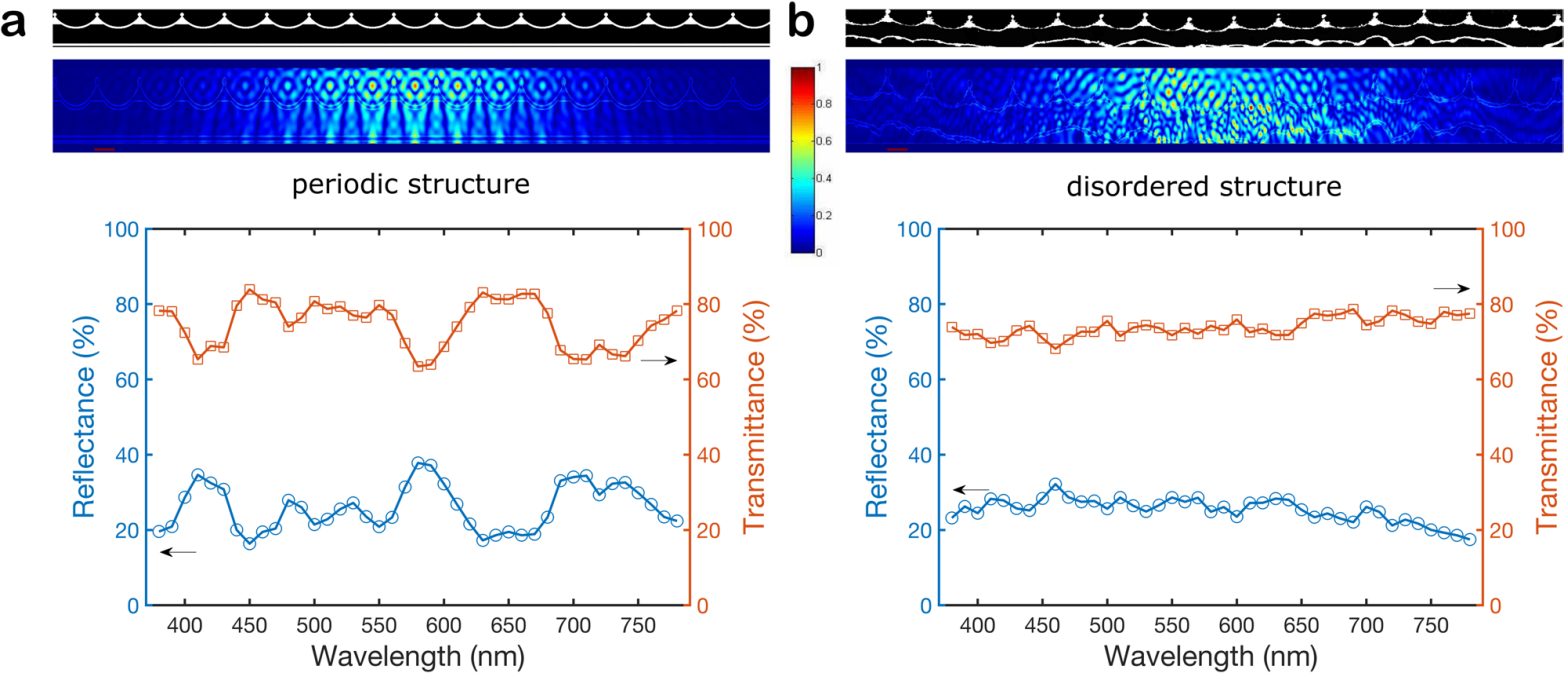
Comparison of the optical response of the **(a)** perfectly periodic structure based on the ’circus tent’ model shown in Fig. 3d and of a **(b)** ’disordered’ structure extracted from an electron microscopy image of a transversal cut. The top images correspond to the black and white images used as structure models in the simulation (black corresponds to air and white to the material of the scale). The middle images show the respective near field obtained for $\lambda = 550$ nm. Red and blue colours correspond to maximum and minimum intensity, respectively; the structure profile is plotted in light blue. The colourbar in the middle corresponds to both near fields. The graphs at the bottom show the respective simulated spectra of reflectance and transmittance.

The top panel of Fig. 5a shows the scheme of this periodic model used to obtain the simulated optical response, the middle panel displays the corresponding near field distribution for $\lambda = 550$ nm. Please see Video S2 for the field distributions at other wavelengths. Red and blue zones correspond to maximum and minimum intensity, respectively; the structure profile is plotted in light blue. As expected, the field distribution is highly symmetric, with a clear intensification in the central zone in which the incident beam is focused. The bottom panel of Fig. 5a shows the simulated reflectance and transmittance of this periodic structure. The reflectance presents a rough profile with several peaks, and takes values between 20 and 40%. Taking into account the transmittance curve, we calculated that conservation of energy is satisfied with an error lower than 6% approximately.

The reflectance and transmittance curves at the bottom of Fig. 5a are typical for periodic systems, and the specific shape of the spectra strongly depends on geometrical characteristics such as the profile’s shape, period, grooves’ depth, thicknesses of the layers and separation between them. It can be noted that the simulation shown in Fig. 5a does not reproduce the features of the measured response presented in Fig. 2c. Unlike the reflectance obtained in the above numerical simulation, which exhibits marked peaks, the experimental reflectance is uniform along the spectrum’s range investigated. This means that the perfectly periodic model does not adequately represent the real structure of the silvery scale. Therefore, the question that arises is: which are the characteristics of the real scale structure that give rise to the measured response? On one hand, as seen in the microscopy images, the upper corrugated layer is not strictly periodic, there are slight variations in shape and thickness between the different grooves and the inclination also changes along the structure. Moreover, microscopy images show that the thickness of the air layer does not remain uniform throughout the scale, and therefore, this could significantly affect the optical response.

Consequently, these irregularities must be taken into account in the model used for simulation. To do so, we consider a cut-out of an electron microscopy image of the cross-section of the scale as a scattering structure within the photonic simulation method. Also in this case, the structure is illuminated by a Gaussian beam of width $\approx 13$ $\upmu$m. At the top of Fig. 5b we show the cross section of a scale extracted from the electron microscopy image, which was used to obtain the near and far fields. As expected, the near field distribution for $\lambda = 550$ nm is much more complex than in the periodic case. Please see Video S3 for the field distributions at other wavelengths. At the bottom of Fig. 5b we show the reflectance and transmittance corresponding to the scale structure extracted from the electron microscopy image. We observe a quite uniform reflectance of approximately 20%, which agrees very well with the experimental measurements of a single silvery scale, as shown in Fig. 2c.

## Conclusion

To conclude, our experimental analysis of the silvery spots of *D. vanillae* shows that they consist of densely packed ground- and coverscales. Our simulations evidence that their metallic appearance is caused by broadband reflections of the disordered three layer system which consists of upper and lower lamina and the air layer in between. This feature causes a uniform reflectance of light for all wavelengths in the visible range of about 20% for ground- as well as for coverscales. Electron microscopy images revealed the inner structure of the scales which can be roughly described by a ’circus tent’ like upper and a flat lower lamina. Subsequent simulations of this inner structure demonstrate that a moderate structural disorder is important for the uniform reflection of light. A periodic model cannot describe the observed spectra, this is only possible with a disordered structure like the one directly extracted from electron microscopy images. With this structure we achieve reasonable agreement between experiment and simulation for the optical spectra of single scales.

Similar to what we report here on the Gulf Fritillary, the lower lamina of *Junonia atlites* was found to generate a macroscopic light gray effect from scales that are actually multicoloured when looked closely as the result of variations in the thickness of the lower lamina^40^. We conclude that randomness in the colour producing structures of butterflies’ wings is key for the production of intense metallic structural colours in wing butterflies^41–43^. Structural colour in butterflies’ wings can evolve fast on the evolutionary time scale as the result of changes in the lower lamina of scales^44^. It is likely that evolutionary rapid colour transitions are the result of a common tuning mechanism allowing to adjust the amount of cuticular secretions during scale formation among Lepidoptera^40,45^. These same mechanisms could contribute to randomness of lower and upper lamina as well as their distance during scale formation.

## Methods

### Samples

All samples of *D. vanillae* used in this study were kindly provided by the Museo Argentino de Ciencias Naturales “Bernardino Rivadavia” collected in several localities within Argentina between 2010 and 2016.

### Optical spectroscopy

Optical spectra of the wing spots were recorded with an UV–Vis–NIR Spectrophotometer (LAMBDA, PerkinElmer) with an integrating sphere. All measurements were normalized with a diffuse white reflectance standard from Labsphere. The diameter of the illuminated area with this set-up is about 2 mm and spans over several scales.

The reflection on single scales was measured with a modular spectrometer (Red Tide USB650, Ocean Optics) coupled to optical microscopes (Leitz Metalloplan and Leica INM 200 UV) with a white lamp (LED100 Cool, Merzhäuser). A thin reflective mirror (68079 OSR CMX 150 from QIOPTIQ) served as reference. With this set-up we can measure single spots allowing for the collection of the reflection of single scales. As shown in Fig. S1, however, the amount of back-scattered light collected by the objective lens depends on the actual magnification. The spectra presented in Fig. 2 were recorded with a 40$\times$ objective.

### Electron microscopy

The scanning electron microscopy images shown in Fig. 3b were recorded with a Zeiss SUPRA 60 VP. Before that the samples were sputtered with a MED 010 from Balzers Union with silver at 100 mA for 50 s to obtain a conductive silver coating of about 20 nm thickness.

To analyse transversal sections of scales as shown in Fig. 3c,e, small pieces of wings were embedded in epoxide resin (Epon: 42.4 g glycid ether 100, 29.6 g DDSA, 18.4 g MNA, and 2.4 g BDMA, all from SERVA). First, wing pieces were immersed in dried acetone for several minutes, followed by 30 min in a mixture of 50% acetone and 50% Epon. Samples were then placed in silicone molds, covered with 100% Epon and polymerized at 60$^\circ$ C for 2 days. From the resulting resin blocks 80 nm thick sections were cut using an ultramicrotome (RMC Boeckeler Powertome PC) and deposited on a piece of silicon wafer. Scanning electron microscope (Ultra 55, Carl Zeiss Microscopy) images of these cross-sections were generated at 1.5 KeV primary electron energy using the InLens detector for secondary electrons and the Atlas 5 software for automated recording of large scan fields. The images used to determine the parameters summarized in Table 1 were recorded with a pixel size of 10 nm.

The measurements of structural features were conducted with the software Image J (version 1.45s).

### Simulations

The calculations of the reflectance of the simplified three-layer-system have been conducted and visualized by using the MATLAB version R2016b from MathWorks^®^.

The applied simulation technique shown in Fig. 5 is inspired by the propagation of mechanical waves along a two-dimensional array of particles contained in the *x–y* plane. Each particle is joined to its four nearest neighbours by means of elastic springs. The movement of the particles is constrained to the *z*-axis, which is normal to the plane of the two-dimensional array along which the waves propagate. A wave is generated by applying an external force along the *z*-axis to certain particles. For a large number of particles, the array can be regarded as a continuous medium representing a tensioned elastic membrane with mass density $\mu$. In analogy with optics, regions with mass density $\mu _0$ can be identified with vacuum, i.e., a medium of refraction index $n_0=1$, whereas a region with an arbitrary mass density $\mu$ corresponds to a medium with a real part of the refraction index $n=\sqrt{\mu /\mu _0}$. This approach can be applied to investigate the optical response of two-dimensional configurations involving dielectric materials, illuminated by transverse electric (TE) polarized light. More details can be found in Refs.^38,39^. It is an advantage of this approach that the simulation domain can be defined by means of digital images or bitmaps, which is very useful to calculate the interaction of light with biological structures.

## Supplementary Information

Supplementary Figures.

Supplementary Video 1.

Supplementary Video 2.

Supplementary Video 3.
