# Supplementary material for: Analysis of the optical properties of the silvery spots on the wings of the Gulf Fritillary, Dione vanillae
Source: Sci Rep. 2021 Sep 29;11:19341. doi: 10.1038/s41598-021-98237-9 (PMC8481520; doi:10.1038/s41598-021-98237-9)
Supplement: Supplementary file 1 — Supplementary Figures. [file 41598_2021_98237_MOESM1_ESM.pdf]

# Supplemental: Analysis of the optical properties of the silvery spots on the wings of the Gulf Fritillary, *Dione vanillae*

**Andrés Dolinko[1,2], Luisa Borgmann[3], Christian Lutz[3], Ernest Ronald Curticean[4], Irene Wacker[4], María Sol Vidal[5], Candela Szischik[5], Yidenekachew Donie[6], Marina Inchaussandague[5,7], Diana Skigin[5,7], Hendrik Hölscher[3,*], Pablo Tubaro[8], and Ana Barreira[8]**

[1]CONICET, Consejo Nacional de Investigaciones Científicas y Técnicas, Argentina
[2]Universidad de Buenos Aires, Facultad de Ciencias Exactas y Naturales, Departamento de Biodiversidad y Biología Experimental, Ciudad Universitaria, Pabellón 2,C1428EHA Buenos Aires, Argentina
[3]Institute for Microstructure Technology, Karlsruhe Institute of Technology (KIT), Hermann-von-Helmholtz-Platz 1, 76344 Eggenstein-Leopoldshafen, Germany
[4]CryoEM, BioQuant, University of Heidelberg, Im Neuenheimer Feld 267, 69120 Heidelberg, Germany
[5]Universidad de Buenos Aires, Facultad de Ciencias Exactas y Naturales, Departamento de Física, Grupo de Electromagnetismo Aplicado, Ciudad Universitaria, Pabellón 1, C1428EHA Buenos Aires, Argentina
[6]Light Technology Institute (LTI), Karlsruhe Institute of Technology (KIT), Engesserstrasse 13, 76131 Karlsruhe, Germany.
[7]CONICET, Universidad de Buenos Aires, Instituto de Física de Buenos Aires (IFIBA), Ciudad Universitaria, Pabellón 1, C1428EHA Buenos Aires, Argentina
[8]División de Ornitología, Museo Argentino de Ciencias Naturales "Bernardino Rivadavia" MACN-CONICET, Av. Angel Gallardo 470 (C1405DJR), Buenos Aires, Argentina
[*]hendrik.hoelscher@kit.edu

## ABSTRACT

Supplemental material of the article including videos and figures.

## Supplemental Videos

**Video S1:** Video of the ventral forewing of a butterfly *Dione vanillae* mounted in rotatable sample holder. The metallic reflection of the silvery spots fade slightly during rotation but the overall colour impression stays the same.
**Video S2:** Animation of the near field calculated for the periodic 'circus tent' model for wavelengths between 380 and 780 nm (in steps of 10 nm). The result for $\lambda = 550$ nm is shown in Fig. 5(a).
**Video S3:** Animation of the near field calculated for the disordered structure extracted from an electron microscopy image for wavelengths between 380 and 780 nm (in steps of 10 nm). The result for $\lambda = 550$ nm is shown in Fig. 5(b).

## Supplemental Figures

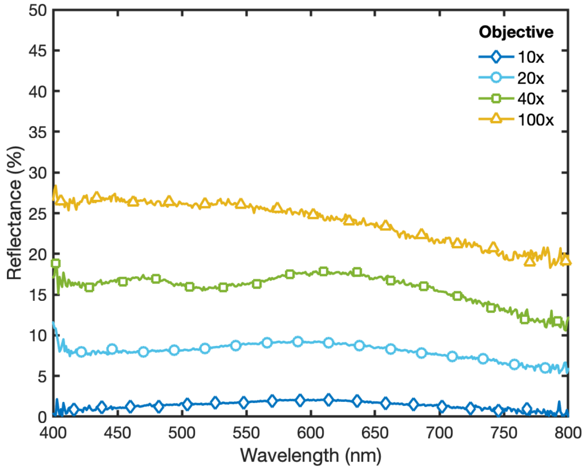


**Figure S1.** Optical microspectroscopy of the upper (external) side of a silvery coverscale measured with different objective lenses ranging from 10x to 100x. Depending on the magnification of the lens the distance to the sample varies. It is shorter for larger magnifications and more light is scattered back into the lens and finally coupled back to the modular spectrometer. This result partly explains the different values for the broadband reflectance obtained for single scales in such a microspectrometer (Fig. 2(c)) and the reflectance values obtained in an integrating sphere on silvery spots (Fig. 1(d)).

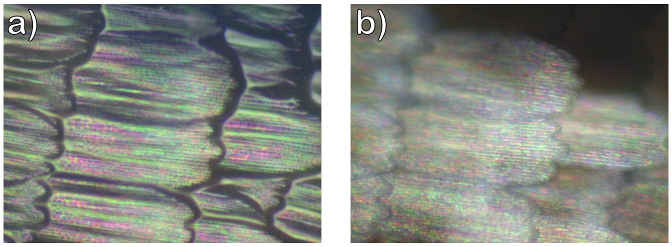


**Figure S2. a)** Photo of the silvery spot soaked with isopropanol ($n \approx 1.38$) taken in an optical microscope. The colour impression shifts from silvery to greenish. **b)** Photo taken at the same position after some minutes of drying when the silvery colour of the scales recovered.
